# Updated analysis of pathogenic variants in *BRCA1*/*BRCA2* among the general Japanese population

**DOI:** 10.1038/s41439-026-00335-5

**Published:** 2026-01-21

**Authors:** Tasuku Mariya, Masashi Idogawa, Tsuyoshi Saito, Hiroshi Nakase, Takashi Tokino, Akihiro Sakurai

**Affiliations:** 1 Division of Clinical Genomics, Department of Genomic and Preventive Medicine, Sapporo Medical University School of Medicine, Sapporo, Japan; 2 Department of Obstetrics and Gynecology, Sapporo Medical University School of Medicine, Sapporo, Japan; 3 Division of Medical Genome Sciences, Department of Genomic and Preventive Medicine, Sapporo Medical University School of Medicine, Sapporo, Japan; 4 Division of Gastroenterology and Hepatology, Department of Internal Medicine, Sapporo Medical University School of Medicine, Sapporo, Japan; 5 Genome Medical Center, Carres Memorial Hospital, Sapporo, Japan

**Keywords:** Genetic variation, Rare variants

## Abstract

Recently, the Tohoku Medical Megabank Organization released whole-genome allele frequencies of single-nucleotide variants and indels from approximately 60,000 individuals from the general Japanese population in the Tohoku region (60KJPN). Here we analyzed the 60KJPN dataset for *BRCA1*/*BRCA2* variants and compared them with the previous version, 54KJPN, to ascertain the frequency of hereditary breast and ovarian cancers in the general Japanese population. We hope that these results will contribute to strategies for cancer prevention.

In a previous paper, we analyzed the whole-genome sequence data of approximately 54,000 individuals from the general population of the Tohoku area in Japan (54KJPN); these data were released by the Tohoku Medical Megabank Organization (ToMMo)^1,2^. We comprehensively examined pathogenic and truncating variants in *BRCA1*/*BRCA2* and their frequency^3^. Recently, ToMMo released an updated data version, including approximately 60,000 individuals (60KJPN). Therefore, we also updated our analysis with 60KJPN and compared it with that of 54KJPN.

To confirm the frequencies of the pathogenic variants, we analyzed ToMMo-60KJPN data (https://jmorp.megabank.tohoku.ac.jp/downloads/tommo-60kjpn-20240904-af_snvindelall) in the same manner as in the previous paper^3,4^. Compared with those in 54KJPN, the number of unique variants of *BRCA1* and *BRCA2* increased from 7711 and 6320 to 8427 and 6885, respectively, in 60KJPN. Among the variants, 44 in *BRCA1* and 66 in *BRCA2* were predicted to be pathogenic or likely pathogenic according to the ClinVar criteria (20250421), and 4 in *BRCA1* and 8 in *BRCA2* were newly identified in 60KJPN (Table 1 and Supplementary Tables 1 and 2). Furthermore, among the truncating variants unclassified in ClinVar, 3 in *BRCA1* and 14 in *BRCA2* were classified as pathogenic on the basis of the ClinGen ENIGMA (Evidence-based Network for the Interpretation of Germline Mutant Alleles) BRCA1 and BRCA2 Expert Panel Specifications to the ACMG/AMP Variant Interpretation Guidelines Version 1.2.0^5^, and 3 in *BRCA2* were newly identified in 60KJPN (Table 1 and Supplementary Tables 1 and 2). The pathogenicity of the three *BRCA1* variants included in 54KJPN changed to likely pathogenic according to the ClinVar update (Table 1). The allele frequencies were more than 1.5 times greater for five pathogenic variants, BRCA1:c.68_69del (p.E23Vfs*17), BRCA2:c.3859_3860del (p.N1287*), c5722_5723del (p.L1908Rfs*2), c.6405_6409del (p.N2135Kfs*3) and c.6715G>T (p.E2239*), in 60KJPN than in 54KJPN. Therefore, we calculated carrier frequencies for *BRCA1* and *BRCA2* by summing all allele counts, assuming each variant occurs independently, assuming each variant occurs independently. Given the very low allele frequencies, the likelihood that an individual carries more than one variant was considered negligible. The estimated carrier frequency in 60KJPN was 2.668 × 10^−3^ (160/119,860.5) for *BRCA1*, 3.219 × 10^−3^ (193/119,807.6) for *BRCA2*, and 5.879 × 10^−3^ for *BRCA1* or *BRCA2* with ClinVar-classified pathogenic variants. The frequency increased to 2.735 × 10^−3^ (164/119,859.3) for *BRCA1*, 3.436 × 10^−3^ (206/119,814.2) for *BRCA2*, and 6.161 × 10^−3^ for *BRCA1* or *BRCA2* with pathogenic variants, including ENIGMA-classified variants.

**Table 1:** Newly recognized pathogenic variants and truncating variants of *BRCA1*/*BRCA2* and their frequencies in the general Japanese population (ToMMo: 60KJPN).

						ToMMo: 60KJPN		gnomAD Exomes v4.1		gnomAD Genomes v4.1		
Gene	cDNA	Amino acids	Exon No.	Type	dbSNP	Allele count	Allele frequency	Total	East Asian	Total	East Asian	Pathogenicity
*BRCA1*	c.121C>G^a^	p.H41D	3	Missense	rs1060502353	1/119,880	8.342 × 10^−6^					Likely pathogenic
	c.1277_1292del	p.S426Yfs*10	10	Frameshift	rs2053962732	1/119,872	8.342 × 10^−6^					Pathogenic
	c.2513del	p.N838Tfs*8	10	Frameshift	rs80357863	1/119,798	8.347 × 10^−6^					Pathogenic
	c.3021_3058del	p.M1008Kfs*7	10	Frameshift	–	1/119,814	8.346 × 10^−6^					Pathogenic
	c.3196G>T	p.E1066*	10	Nonsense	rs1555588220	1/119,826	8.345 × 10^−6^					Pathogenic
	c.5145C>A^a^	p.S1715R	17	Missense	rs80357094	1/119,874	8.342 × 10^−6^					Likely pathogenic
	c.5257A>G^a^	p.R1753G	19	Missense	rs1597810544	1/119,880	8.342 × 10^−6^					Likely pathogenic
*BRCA2*	c.124_128del	p.Y42Ffs*2	3	Frameshift	rs786201783	1/119,880	8.342 × 10^−6^					Pathogenic (E)
	c.475+1G>A		5	Splicing	rs81002797	1/119,880	8.342 × 10^−6^					Pathogenic
	c.2034_2041del	p.T680Lfs*5	11	Frameshift	–	1/119,880	8.342 × 10^−6^					Pathogenic (E)
	c.3650del	p.G1218Afs*10	11	Frameshift	–	1/119,754	8.350 × 10^−6^					Pathogenic (E)
	c.4627del	p.N1544Tfs*24	11	Frameshift	rs80359460	1/119,684	8.355 × 10^−6^	4.106 × 10^−6^	4.106 × 10^−6^	1.315 × 10^−5^	2.520 × 10^−5^	Pathogenic
	c.5106_5109del	p.R1704*	11	Nonsense	rs879254123	1/119,710	8.354 × 10^−6^	1.394 × 10^−6^	1.394 × 10^−6^			Pathogenic
	c.5638_5641del	p.K1881Qfs*27	11	Frameshift	rs276174860	1/119,748	8.351 × 10^−6^					Pathogenic
	c.6359C>A	p.S2120*	11	Nonsense	–	1/119,832	8.345 × 10^−6^					Pathogenic
	c.6385G>T	p.E2129*	11	Nonsense	rs886040653	1/119,832	8.345 × 10^−6^	6.879 × 10^−7^	6.879 × 10^−7^			Pathogenic
	c.7558C>T	p.R2520*	15	Nonsense	rs80358981	1/119,880	8.342 × 10^−6^	6.840 × 10^−6^	6.840 × 10^−6^	1.314 × 10^−5^		Pathogenic
	c.8970G>A	p.W2990*	23	Nonsense	rs80359149	1/119,858	8.343 × 10^−6^					Pathogenic

Variant annotations are based on BRCA1 (NM_007294.4) and BRCA2 (NM_000059.4). Pathogenicity is based on ClinVar (20250421). Truncating variants without ClinVar pathogenicity are evaluated by the ENIGMA criteria (E). Variants are considered pathogenic when they correspond to PVS1 + PM5_PTC.

^a^Although these variants are included in ToMMo: 54KJPN, the pathogenicity was changed to likely pathogenic by the ClinVar update.

In the analysis of 60KJPN, a total of 15 pathogenic variants in *BRCA1*/*BRCA2* were newly identified, and all estimated carrier frequencies were higher than those in 54KJPN, even though the sample size increased by only 6000 individuals (12,000 alleles) from 54KJPN to 60KJPN. These results suggest the possibility of other rare pathogenic variants in the general Japanese population.

In addition, many variants of uncertain significance (VUSs) were also identified in *BRCA1*/*BRCA2* as rare variants in the 60KJPN dataset; VUSs may represent yet-undetermined, population-specific pathogenic variants. Notably, two recent intriguing reports concerning BRCA2 focused on the DNA-binding domain (DBD), where ClinVar-registered VUSs are enriched^6,7^. This DBD plays a critical role in the homologous recombination function of BRCA2 through single-stranded DNA binding and RAD51 recruitment, making it highly sensitive to functional disruption by missense or truncating variants. In addition, the moderate size and well-defined structural features of the domain allow feasible saturation genome editing and robust phenotypic readouts in cell-based assays. In their respective studies, Sahu et al. used saturation genome editing in mouse embryonic stem cells, whereas Huang et al. applied a CRISPR–Cas9–based saturation genome editing approach in haploid human cells, together providing a comprehensive, experimentally derived reference for the clinical interpretation of BRCA2 DBD variants. When cross-referenced with variants detected in 60KJPN, among the 63 variants in the DBD classified in ClinVar as VUSs or with conflicting interpretations of pathogenicity, 49 were rated as likely benign or benign in both studies, and 9 were rated as likely benign in either study (Supplementary Table 3). Thus, most of the variants were classified as likely benign or benign. Among the remaining variants, two remained as VUSs in both studies, and three were rated as likely pathogenic in either study; however, no variants were consistently classified as likely pathogenic/pathogenic in both analyses. Because many variants remain classified as VUS under ENIGMA criteria, the impact of saturation genome editing-based functional assessment is particularly substantial. Of note, among ClinVar-unclassified variants, nine were classified as Likely Benign or Benign by ENIGMA criteria, and all of these classifications were concordant with the results of saturation genome editing studies. Furthermore, for variants classified as benign or pathogenic in ClinVar, the results of these analyses revealed nearly identical classifications. Thus, these comprehensive approaches using saturation genome editing have the potential to elucidate the pathogenicity of unresolved VUSs in other domains and genes in future studies.

The allele count of two pathogenic variants, BRCA1:c.4601dup (p.E1535Gfs*39) and BRCA2:c.6666C>G (p.Y2222*), changed from one in 54KJPN to zero in 60KJPN. Inquiries with ToMMo revealed that this change is due to the exclusion of close relatives in each dataset, aimed at preventing an upward shift in allele frequencies caused by the accumulation of related individuals, which may have led to differences in the samples used compared with previous versions. Therefore, care should be taken when evaluating rare variants.

Since ToMMo announced that it has already acquired genome data for approximately 100,000 people, it is expected that the analyzed data will be available in the near future. Expanding the sample size is expected to contribute not only to a more comprehensive understanding of the diversity of *BRCA1*/*BRCA2* pathogenic variants but also to cancer prevention and treatment decisions in Japan.

## Supplementary information

Supplementary Table 1. Pathogenic variants and truncating variants of BRCA1 and their frequencies in the general Japanese population (ToMMo: 60KJPN).

Supplementary Table 2. Pathogenic variants and truncating variants of BRCA2 and their frequencies in the general Japanese population (ToMMo:60KJPN).

Supplementary Table 3. Pathogenicity evaluation of BRCA2 DBD variants and their frequencies in the Japanese general population (ToMMo: 60KJPN).
